# Circulating MACC1 Transcripts in Colorectal Cancer Patient Plasma Predict Metastasis and Prognosis

**DOI:** 10.1371/journal.pone.0049249

**Published:** 2012-11-14

**Authors:** Ulrike Stein, Susen Burock, Pia Herrmann, Ina Wendler, Markus Niederstrasser, Klaus-Dieter Wernecke, Peter M. Schlag

**Affiliations:** 1 Experimental and Clinical Research Center, Charité University Medicine Berlin, at the Max-Delbrück-Center for Molecular Medicine, Berlin, Germany; 2 Charité Comprehensive Cancer Center, Berlin, Germany; 3 Institute of Medical Biometry, Charité University Medicine Berlin, Berlin, Germany; Howard University, United States of America

## Abstract

**Background:**

Metastasis is the most frequent cause of treatment failure and death in colorectal cancer. Early detection of tumors and metastases is crucial for improving treatment strategies and patient outcome. Development of reliable biomarkers and simple tests routinely applicable in the clinic for detection, prognostication, and therapy monitoring is of special interest. We recently identified the novel gene Metastasis-Associated in Colon Cancer 1 (MACC1), a key regulator of the HGF/Met-pathway. MACC1 is a strong prognostic biomarker for colon cancer metastasis and allows identification of high-risk subjects in early stages, when determined in patients’ primary tumors. To overcome the limitation of a restricted number of molecular analyses in tumor tissue, the establishment of a non-invasive blood test for early identification of high-risk cancer patients, for monitoring disease course and therapy response is strongly needed.

**Methodology/Principal Findings:**

For the first time, we describe a non-invasive assay for quantification of circulating MACC1 transcripts in blood of more than 300 colorectal cancer patients. MACC1 transcript levels are increased in all disease stages of the cancer patients compared to tumor-free volunteers. Highest MACC1 levels were determined in individuals with metastases (all P<0.05). Importantly, high MACC1 levels correlate with unfavorable survival (P<.0001). Combining MACC1 with circulating transcripts of the metastasis gene S100A4, a transcriptional target of the Wnt/β-catenin-pathway, improves survival prediction for newly diagnosed cancer patients.

**Conclusion/Significance:**

This blood-based assay for circulating MACC1 transcripts, which can be quantitated on a routine basis, is clinically applicable for diagnosis, prognosis, and therapeutic monitoring of cancer patients. Here we demonstrate the diagnostic and prognostic value of circulating MACC1 transcripts in patient plasma for metastasis and survival. Since MACC1 represents a promising target for anti-metastatic therapies, circulating MACC1 transcripts may prove to be an ideal read-out for monitoring therapeutic response of future interventions targeting MACC1-induced metastasis in cancer patients.

## Introduction

Metastasis is the most frequent cause of treatment failure and death in colorectal cancer. Early detection of tumors and metastases is crucial for improving treatment strategies and patient outcome. Development of reliable biomarkers and simple tests that are routinely applicable in the clinic for detection, prognostication, and therapy monitoring is of special interest.

We recently identified the novel gene Metastasis-Associated in Colon Cancer 1 (MACC1) [1]–[3]. MACC1 is a new prognostic biomarker for colon cancer metastasis and metastasis-free survival when determined in patients’ primary tumors. MACC1 levels in the primary tumors were found to be significantly higher in cancers that metachronously developed distant metastases compared to those which did not metastasize within a 12-year follow-up. The predictive value of the biomarker on whether a tumor will metastasize or not was 74% and 80% respectively. The 5-year-survival rate was 80% for patients with low MACC1, compared to 15% for patients with high MACC1 expression in their primary tumors. MACC1 is a predictor for colorectal cancer metastasis independent of tumor stage, age, sex, tumor infiltration, nodal status and lymph vessel invasion, and thus allows identification of subjects at high risk for metastasis in early stages [1]. This makes MACC1 an important prognostic gene in clinical practice. Furthermore, we found that MACC1 acts a key regulator of the HGF (hepatocyte growth factor)/Met-pathway, which is crucial in colorectal cancer for tumor progression and metastasis formation [1], [2], [4].

Several groups confirmed meanwhile a correlation of high MACC1 expression in the tumor to progression, metastasis, survival, and therapy response in colorectal cancer (CRC) [5]–[9]. The role of MACC1 as a biomarker for cancer progression and survival was meanwhile also reported for other solid cancers, such as gastric [10], lung [11], [12], hepatocellular [13], [14] and ovarian cancer [15].

Molecular analyses in tumor tissue, e.g. needed for disease prognosis in the context of metastasis formation and survival, are limited to a restricted number of available tissue samples. To overcome this limitation, the establishment of a non-invasive MACC1-based blood test for early identification of high risk cancer patients and for monitoring of the disease course as well as of therapy response is therefore strongly needed.

Here, for the first time, we developed a non-invasive assay for MACC1 transcript levels in plasma. We analyzed the diagnostic value of MACC1 levels for detection of tumors and metastases in colon and rectal cancer patients, and its prognostic value for overall survival (survival) of these patients. Furthermore, we evaluated the benefit of combining MACC1 with a further metastasis gene, S100A4, for improved prediction of disease prognosis [16]–[18].

## Materials and Methods

### Objective

The metastasis-inducing gene MACC1 is a strong prognostic biomarker for colon cancer metastasis and allows identification of high-risk subjects in early stages, when determined in patients’ primary tumors. To overcome the limitation of a restricted number of molecular analyses in tumor tissue, we aimed at the establishment of a non-invasive MACC1-based blood test for early identification of high risk cancer patients and for monitoring the disease course.

### Participants

For colon and rectal cancer patients’ characteristics see Tables 1, 2, 3. Consecutive patients with colon or rectal cancer who were seen at the Robert Rössle Cancer Hospital, Charité University Medicine Berlin, during 2006 until 2007 for inpatient or outpatient care and who gave their written informed consent to take part in our tumor biobank were enrolled.

**Table 1:** Colorectal cancer patients characteristics and circulating MACC1 transcript levels in plasma.

	Tumor-freevolunteers	Newly diagnosedprimary tumorwithout metastasis	Newly diagnosedprimary tumorwith synchronousmetastasis	Newly diagnosedmetachronousmetastasis	Follow-up	All cancerpatients
Blood samples, n	54	51	20	11	230	312
UICC I, %		12	0	18	27	22
UICC II, %		27	0	0	27	24
UICC III, %		61	0	72	45	46
UICC IV, %		0	100	9	2	8
Adjuvant therapy		15/51	–	6/11	129/230	150/312
Follow up, median, days		850	461	711	840	840
Age, median (range), years	61 (27–87)	68 (19–84)	65 (38–78)	64 (48–74)	63 (18–81)	64 (18–84)
Sex, male/female	43/11	35/16	14/6	6/5	145/85	200/112
MACC1 mRNA expression,% calibrator (median)	0.224	0.610	0.937	0.700	0.490	0.520
*P*, vs tumor-free volunteers		<.001	<.001	<.001	<.001	<.001

**Table 2:** Colon cancer patients characteristics and circulating MACC1 transcript levels in plasma.

	Tumor-freevolunteers	Newly diagnosedprimary tumorwithout metastasis	Newly diagnosedprimary tumorwith synchronousmetastasis	Newly diagnosedmetachronousmetastasis	Follow-up	All cancerpatients
Blood samples, n	54	12	10	4	125	151
UICC I, %		42	0	0	26	25
UICC II, %		42	0	0	27	26
UICC III, %		7	0	75	43	39
UICC IV, %		0	100	25	3	10
Adjuvant therapy		2/12	–	1/4	50/125	53/151
Follow up, median, days		935	302	590	807	805
Age, median (range), years	61 (27–87)	66 (51–75)	67 (38–78)	59 (48–62)	63 (34–81)	63 (34–81)
Sex, male/female	43/11	10/2	6/4	1/3	71/54	88/63
MACC1 mRNA expression,% calibrator (median)	0.224	0.780	2.249	0.968	0.504	0.516
*P*, vs tumor-free volunteers		<.001	<.001	.016	<.001	<.001

**Table 3:** Rectal cancer patients characteristics and circulating MACC1 transcript levels in plasma.

	Tumor-freevolunteers	Newly diagnosedprimary tumorwithout metastasis	Newly diagnosedprimary tumorwith synchronousmetastasis	Newly diagnosedmetachronousmetastasis	Follow-up	All cancerpatients
Blood samples, n	54	39	10	7	105	161
UICC I, %		3	0	29	27	19
UICC II, %		23	0	0	27	23
UICC III, %		74	0	71	47	52
UICC IV, %		0	100	0	0	6
Adjuvant therapy		13/39	–	5/7	79/105	97/161
Follow up, median, days		848	994	751	853	818
Age, median (range), years	61 (27–87)	67 (19–84)	63 (59–72)	67 (49–74)	62 (18–77)	64 (18–84)
Sex, male/female	43/11	25/14	8/2	5/2	74/31	112/49
MACC1 mRNA expression,% calibrator (median)	0.224	0.559	0.829	0.621	0.490	0.520
*P*, vs tumor-free volunteers		<.001	<.001	.002	<.001	<.001

Patients’ data for histopathological characterization of the tumor (including tumor infiltration, lymph node status, metastasis, grading, lymphatic vessels infiltration, blood vessels infiltration, residual tumor), for treatment and survival were available from the tumor bank of the Charité Comprehensive Cancer Center. Histological tumor staging was performed by routine pathology. Exclusion criteria were other malignancies during history or follow-up. We analyzed 312 blood samples of colon (n = 151) and rectal (n = 161) cancer patients (Tables 1, 2, 3).

Blood samples were taken at the day of diagnosis from newly diagnosed patients with a primary tumor without or with synchronous metastases or from patients who developed metastases metachronously after R0-resection of the primary tumor. Exception: Patients with newly diagnosed locally advanced rectal cancer, who received neoadjuvant short-course radiation or long-term radiochemotherapy (n = 16). Here, blood samples were taken after neoadjuvant treatment of the primary tumor. For patients receiving short-course radiation, the interval between the end of the treatment and blood taking was 3 to 6 days. We did not find significantly different MACC1 transcript levels between the treated (n = 16) and the non-treated rectal cancer patients (n = 23).

Blood samples from the cohort of follow-up patients were taken exclusively during the follow-up period (median 975 and 1280 days after primary diagnosis; median 807 and 853 days follow-up after blood taking for colon and rectal cancer, respectively).

The plasma controls derived from two independent cohorts of volunteers (n = 54). Recruitment of the volunteers was supported by Klaus Sperber, Medical Practioner, Berlin, and by Ursula Plöckinger, Charité Campus Virchow Klinikum, Berlin. All volunteers must be tumor-free and without a history of oncological diseases. We did not find significantly different MACC1 levels with respect to gender, age, or between the two cohorts.

Newly diagnosed colorectal cancer patients with a primary tumor with or without synchronous metastases were grouped due to their chronological examination in a test-set (n = 36) and a validation-set (n = 35) for determination of the diagnostic value of circulating MACC1 transcripts (Table 4).

**Table 4:** Diagnostic value of circulating MACC1 transcript levels in plasma of newly diagnosed colorectal, colon, and rectal cancer patients with a primary tumor with or without synchronous metastasis.

Cancer	Samples	Optimal cut-off	Sensitivity	Specificity
	n	MACC1 mRNA expression, % calibrator	%	%
Colorectal	71	0.398	76	76
Colon	22	0.503	77	89
Rectal	49	0.398	76	76

Optimal cut offs were calculated with a fourfold table.

### Plasma Preparation

**Figure 1:**
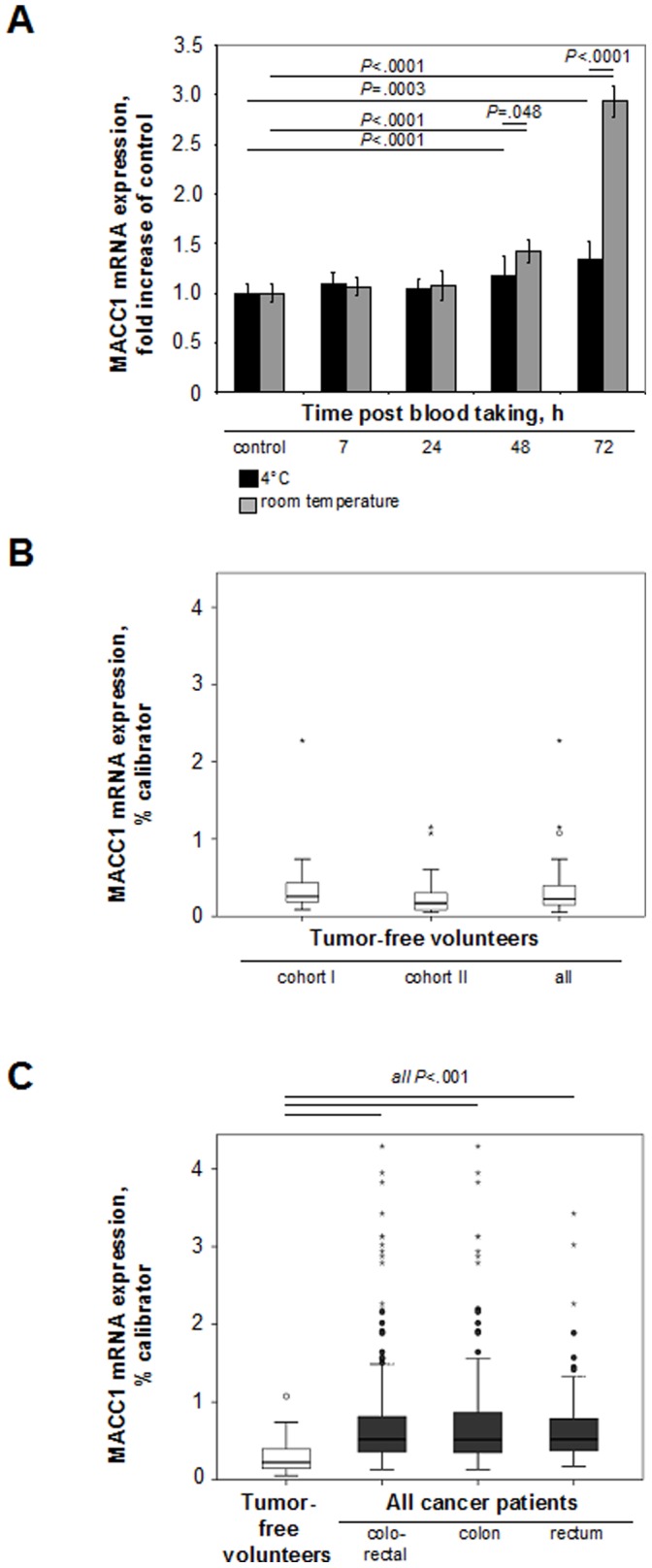
Circulating MACC1 transcript levels in plasma discriminate cancer patients from tumor-free volunteers. A. Plasma separation conditions for determination of circulating MACC1 transcripts. Plasma separation of samples from tumor-free volunteers (n = 12) was done immediately, 7, 24, 48, and 72 hours after taking blood, either from blood samples kept at 4°C or at room temperature. Subsequently, plasma was generated within the first 7 hours from 4°C-cooled blood. B. MACC1 transcripts in plasma of tumor-free volunteers (n = 54). No difference in MACC1 transcript levels were found in two independently analyzed cohorts of tumor-free volunteers (n = 34 and n = 20, respectively). C. MACC1 transcripts in plasma of all colorectal cancer patients (n = 312). All patient cohorts bearing colorectal, colon (n = 151), or rectal (n = 161) cancer expressed significantly higher MACC1 transcript levels than tumor-free volunteers (*P*<.001 for all comparisons).

First, we examined optimal conditions for blood taking, storage, and plasma separation. Following blood taking of healthy volunteers (n = 12), EDTA-blood was kept at room temperature or at 4°C. Plasma separation was done either immediately, or 7, 24, 48, and 72 hours after blood taking (10 analyses per volunteer). RNA was isolated and MACC1-specific quantitative real-time RT-PCR was performed in duplicates (Fig. 1A). MACC1 transcript levels served as read-out. We found no alterations of MACC1 levels when the plasma separation was carried out during the first 24 hours (either cooled or kept at room temperature). Based on these findings, plasma was separated from cooled EDTA-blood at the same day within 7 hours post blood taking. Procedure for plasma separation was as previously described [18]. Blinded samples, neither colon nor rectal cancer, nor disease stage was disclosed during analysis, were stored at −80°C.

### RNA and Quantitative Real-Time RT-PCR

Isolation of total RNA was performed as previously described [18]. Quantitative real-time RT-PCR was carried out at 30 sec 95°C, 45×(10 sec 95°C, 10 sec 62°C, 10 s 72°C), melting curve 40°C to 95°C, by using the LightCycler (DNA Master Hybridization Probes kit, Roche Diagnostics) as previously described [1]. The following primers and probes were used amplifying a 136 bp MACC1-specific PCR product: forward primer 5′-TTCTTTTGATTCCTCCGGTGA-3′, reverse primer 5′-ACTCTGATGGGCATGTGCTG-3′, FITC-probe 5′-GCAGACTTCCTCAAGAAATTCTGGAAGATCTA-3′, LCRed640-probe 5′-AGTGTTTCAGAACTTCTGGACATTTTAGACGA-3′ (syntheses of primers and probes: BioTeZ and TIB MolBiol, Berlin, Germany). The calibrator cDNA was employed in serial dilutions simultaneously in each run, derived from the cell lines SW620. Authentification of the cell line was performed by short tandem repeat (STR) genotyping (DSMZ Braunschweig, Germany). STR genotype was consistent with the published genotype for this cell line (ATCC, CCL-227). S100A4-specific quantitative real-time RT-PCR was carried as previously described [18]. mRNA expression of a blood sample is given as percentage of the mRNA expression of a defined calibrator sample, which was set 100%. Each sample was run in duplicate, the means are depicted.

### Ethics

All blood specimens from patients and tumor-free volunteers were obtained with informed written consent in accordance to the International Conference on Harmonisation and with approval of the local IRB.

### Statistical Methods

Differences between groups in terms of MACC1 transcript levels in plasma were tested by using non-parametric Wilcoxon-Mann-Whitney tests (dependent on the distribution of normality): tumor-free volunteers vs. patients with primary tumors without and with synchronous metastases, to patients with metachronous metastases, and to follow-up patients; patients with tumors vs. those with tumors and metastases. In case of small samples, greater differences in sample sizes, large but unbalanced groups, data sets containing ties, or sparse data, tests were carried out in an exact version. We considered *P*<0.05 to be significant. All numerical calculations were performed with SPSS, version 18.

To define the diagnostic value of circulating MACC1 transcripts in plasma, sensitivity and specificity were calculated with a fourfold table for colorectal cancer (test- and validation-set), colon, and rectal cancer patients, who were newly diagnosed with a primary tumor without or with synchronous metastases compared to the blood samples of 54 tumor-free volunteers. In accordance with their chronological examination and initial blood taking patients were grouped in a test-set and validation-set. We began with a test-set of newly diagnosed CRC patients (n = 36), determined the optimal cut-off value of MACC1 (sensitivity 75%, specificity 76%), and applied this cut-off value for the validation-set of CRC patients (n = 35). Sensitivity and specificity were 77% and 76%, respectively. When combining all newly diagnosed CRC patients, sensitivity and specificity were 76%. When analyzing colon and rectal cancer patients separately, sensitivity was 77% for colon cancer and 76% for rectal cancer. Specificity was 89% for colon cancer and 76% for rectal cancer, respectively.

For survival analysis of newly diagnosed and of all CRC patients, Kaplan Meier curves in combination with log rank test were used. Those CRC patients were included in this analysis where both markers, MACC1 and S100A4, could be determined (n = 294). The cut-off value of MACC1 and the cut-off value of S100A4 were the median of the investigated groups (primary diagnosis or all patients), respectively. Calculations were performed with SPSS, version 18.

## Results

### Quantification of Circulating MACC1 Transcripts in Human Plasma

We initially identified optimal conditions for plasma processing and MACC1 transcript quantification after blood taking. Plasma separation and subsequent MACC1 analyses were performed immediately or 7, 24, 48, and 72 hours after blood taking from tumor-free volunteers (n = 12; 10 aliquots per volunteer), either 4°C-cooled or left at room temperature (Fig. 1A). Increased MACC1 levels were measured due to hemolysis after 48 and 72 hours following taking blood. To determine the basal levels of circulating MACC1 transcripts in tumor-free volunteers and colorectal cancer patients, we subsequently separated plasma from 4°C-cooled blood samples within the first 7 hours after blood taking.

### Circulating MACC1 Transcripts in Plasma of Tumor-free Volunteers

We analyzed the basal MACC1 levels of tumor-free volunteers in two independent cohorts (n = 34 and n = 20, respectively; Fig. 1B). MACC1 transcripts were detected in all samples, without significant differences between the two cohorts. All tumor-free volunteers were combined for subsequent analyses (median 0.224 MACC1 mRNA expression/% calibrator).

### Circulating MACC1 Transcripts in Plasma Discriminate Tumor-free Volunteers and Colorectal Cancer Patients

Next, we analyzed MACC1 levels in plasma of colon (n = 151) and rectal (n = 161) cancer patients (patients’ characteristics: Tables 1, 2, 3). To evaluate the diagnostic value of circulating MACC1 transcripts in plasma, we compared newly diagnosed CRC patients with a primary tumor with or without synchronous metastases (n = 71) with tumor-free volunteers (n = 54) and calculated sensitivity and specificity with a fourfold table. We began with a test-set of newly diagnosed CRC patients (n = 36; sensitivity 75%; specificity 76%), and applied the calculated optimal cut-off value for the validation-set of newly diagnosed CRC patients (n = 35; sensitivity 77%; specificity 76%). When combining all CRC patients newly diagnosed, sensitivity and specificity were 76% (Table 4). When analyzing colon and rectal cancer patients separately, sensitivities were 77% and 76%, and specificities were 89% and 76%, respectively. Thus, MACC1 transcript levels in plasma supports the identification of individuals bearing colon and rectal cancer.

We detected MACC1 transcripts in plasma of all cancer patients. We found significantly higher MACC1 levels in colon, rectal, and colorectal (combined) cancer patients compared to tumor-free individuals (all *P*<.001; Fig. 1C). We observed no significant variations of MACC1 levels due to age, sex, or between the tumor stages I, II and III.

### High Circulating MACC1 Transcript Levels in Plasma of Metastasized Colorectal Cancer Patients

**Figure 2:**
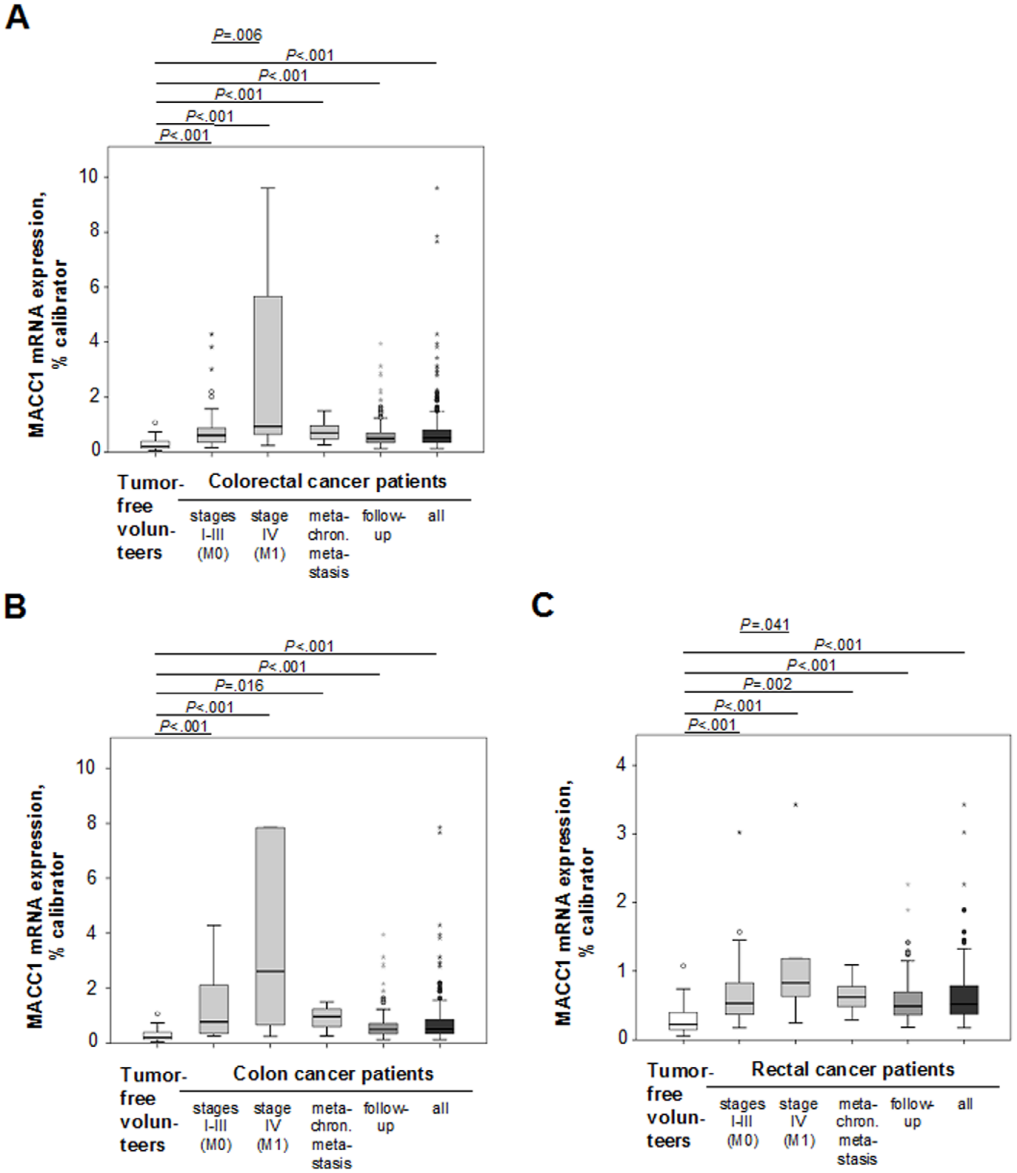
Circulating MACC1 transcripts in colorectal, colon, and rectal cancer patient plasma. A, B, C. All colorectal (A, n = 312), colon (B, n = 151), and rectal (C, n = 161) cancer patient sub-cohorts showed significantly higher circulating MACC1 transcript levels than tumor-free volunteers (n = 54). Higher MACC1 levels were also found for colorectal (*P* = .006; A) and rectal (*P* = .041; C) cancer patients with synchronous metastases (n = 20 and n = 10, respectively) compared to patients without distant metastases (n = 51 and n = 39, respectively). Box plot analysis, based on quantitative real-time RT-PCR.

To evaluate the relevance of circulating MACC1 transcripts in plasma with respect to metastasis formation, patients were classified according to disease stage: patients with a primary tumor without synchronous metastasis (stages I-III; n = 51 for colorectal, n = 12 for colon, and n = 39 for rectal cancer patients); patients with a primary tumor and synchronous metastasis (stage IV; n = 20 for colorectal, n = 10 for colon, and n = 10 for rectal cancer patients); and individuals with metachronous metastasis after R0-resection of the primary tumor (n = 11 for colorectal, n = 4 for colon, and n = 7 for rectal cancer patients) (Tables 1, 2, 3). Blood was taken at the day of diagnosis. Additionally, we also included follow-up patients (n = 230 for colorectal, n = 125 for colon, and n = 105 for rectal cancer patients; Tables 1, 2, 3). MACC1 levels were significantly higher in each disease stage of colon and rectal cancer compared with tumor-free volunteers (Fig. 2A–C). Remarkably, clearly increased MACC1 levels were detected in newly diagnosed cancer patients with synchronous metastasis (stage IV), compared to cancer patients without distant metastases (stages I-III): for colorectal (Fig. 2A), colon (Fig. 2B), and rectal cancer (Fig. 2C), medians 0.937, 2.249, and 0.829 MACC1 mRNA expression/% calibrator in metastasized patients, compared to 0.61, 0.78, and 0.559 MACC1 mRNA expression/% calibrator in patients without distant metastases (*P* = .006, *P* = .176, and *P* = .041 for colorectal, colon, and rectal cancer patients, respectively). Thus, quantitative determination of MACC1 transcripts in patient blood is of diagnostic value with respect to metastasis.

### High Circulating MACC1 Transcript Levels in Plasma of Colorectal Cancer Patients is Associated with Shorter Survival

**Figure 3:**
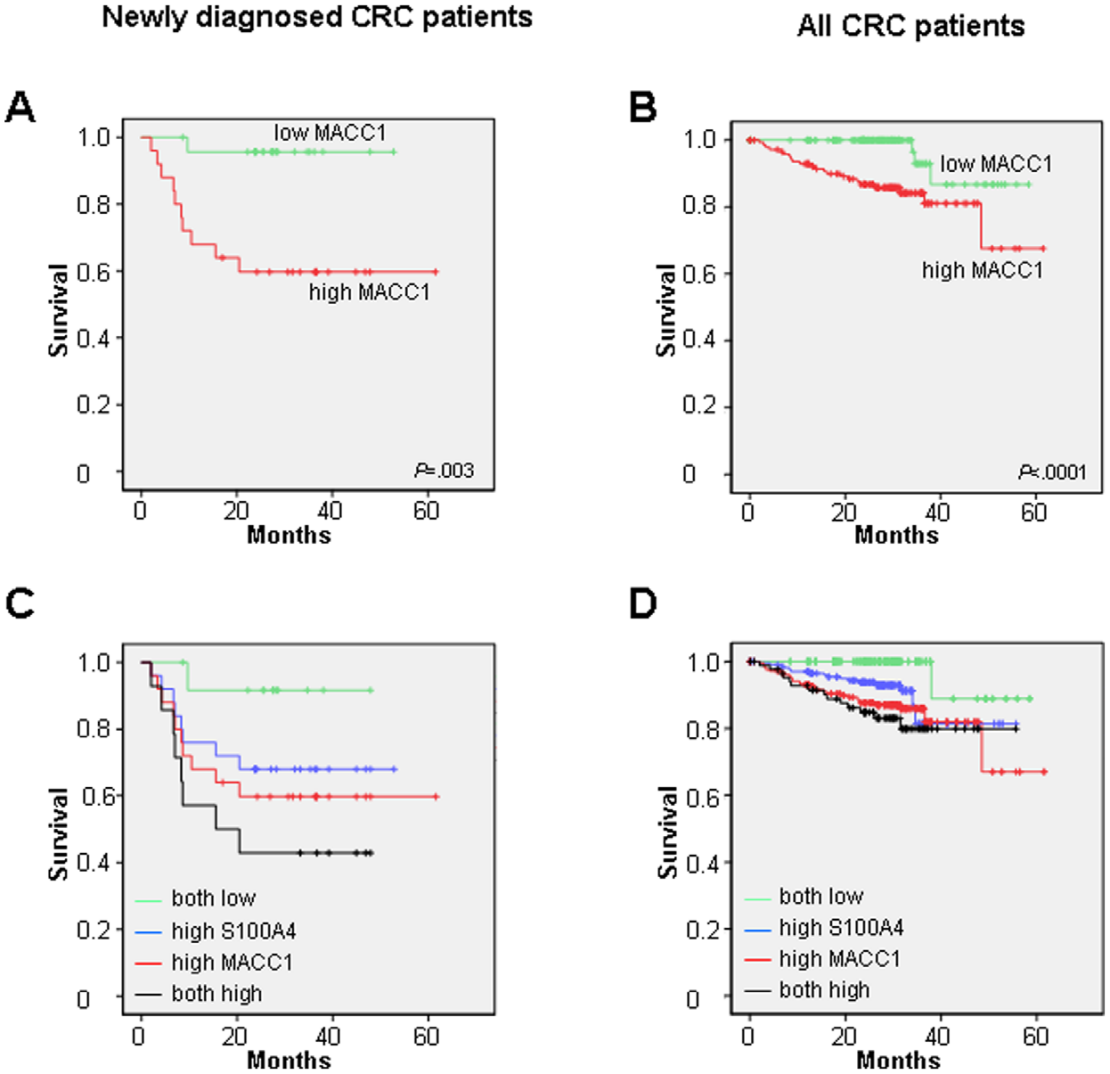
Survival of CRC patients based on circulating transcript levels of MACC1 or a combination of MACC1 and S100A4. A, B. Kaplan-Meier analysis for newly diagnosed (A, n = 49) and all (B, n = 294) CRC patients, based on MACC1 alone (A, low MACC1 n = 24, high MACC1 n = 25; B, low MACC1 n = 147, high MACC1 n = 147). Newly diagnosed CRC patients (A) as well as all CRC patients (B) with high circulating MACC1 transcript levels demonstrated shorter survival when compared with patients demonstrating low MACC1 levels (*P* = .003 and *P*<.0001, respectively). C, D. Kaplan-Meier analysis for newly diagnosed (C, n = 49) and all (D, n = 294) CRC patients, based on a combination of MACC1 and S100A4. Newly diagnosed CRC patients (C) and all CRC patients (D) were classified into groups of low expressors of both genes (n = 13 and n = 85, respectively), of patients with high S100A4 levels (n = 25 and n = 147, respectively), of patients with high MACC1 levels (n = 25 and n = 147, respectively), or with high expression of both biomarkers (n = 14 and n = 85, respectively). For newly diagnosed patients (C), survival was reduced with increasing levels of circulating transcripts of S100A4, of MACC1, or of both. Expression induction of either MACC1 or S100A4 or of both biomarkers correlated to reduced patients survival.

Next, we evaluated the prognostic impact of circulating MACC1 transcripts in plasma. We began the analysis of circulating MACC1 levels with respect to the survival of newly diagnosed CRC patients with a primary tumor with or without synchronous metastases (blood samples were taken at the day of diagnosis) (Fig. 3A). The cut-off value used was the median of the measured MACC1 levels in this patient cohort (0.670 MACC1 mRNA expression/% calibrator). In accordance to this cut-off, patients were classified as low or high MACC1 expressors. Interestingly, those newly diagnosed patients with low MACC1 levels (<cut-off) demonstrated a significantly longer survival time than patients with high MACC1 levels (>cut-off) (*P* = .003; Fig. 3A). Thus, circulating MACC1 transcript levels in plasma when determined at the day of diagnosis, are of prognostic value for the survival of newly diagnosed patients.

Next, we wished to analyze on whether a prognostic impact of MACC1 transcripts holds true for the cohort of all CRC patients, including the follow-up patients. The cut-off value used was the median of the measured MACC1 levels of all CRC patients (0.518 MACC1 mRNA expression/% calibrator). Based on this cut-off, all CRC patients were grouped as low or high MACC1 expressors. Remarkably, we could confirm our finding from the group of newly diagnosed patients also for all CRC patients analyzed: patients with low MACC1 levels (<cut-off) showed a significantly longer survival time than patients with high MACC1 levels (>cut-off) (*P*<.0001; Fig. 3B). Thus, levels of circulating MACC1 transcripts in CRC patient plasma are of prognostic value with respect to patient survival.

### Combination of Circulating MACC1 and S100A4 Transcript Levels in Plasma Improves Survival Prediction for Colorectal Cancer Patients

To further improve prediction of survival, we added the metastasis gene S100A4, a target of the Wnt/β-catenin-pathway, to this MACC1 analysis. We had chosen these metastasis biomarkers for combination, since they address most relevant signaling pathways in colorectal cancer progression and metastasis: MACC1 - a key regulator of the HGF/Met signaling pathway, and S100A4– a transcriptional target gene of the Wnt/β-catenin signaling pathway. So far, no cross regulation of these two metastasis-associated genes - MACC1 and S100A4 - is known.

Furthermore, we previously reported the diagnostic and prognostic value of S100A4 transcripts in plasma [18]. Here, individuals with low S100A4 plasma levels also showed a longer survival (Fig. S1A,B).

We first addressed the question on whether the combination of both biomarkers, circulating transcripts of MACC1 and of S100A4, might improve the prognostic value of MACC1 alone in the cohort of newly diagnosed CRC patients. The cut-off values used were the median of the measured MACC1 levels in this patient cohort (0.670 MACC1 mRNA expression/% calibrator) as well as the median of the measured S100A4 levels in this patient cohort (0.435 S100A4 mRNA expression/% calibrator). In accordance to these cut-off values, patients were classified as low expressors of both genes, as high expressors of S100A4, as high expressors of MACC1, or as high expressors of both genes. Patients who showed low circulating levels of MACC1 and of S100A4, had the longest survival times. Reduced survival was then observed for patients with high S100A4 levels, followed by patients with high MACC1 levels. Shortest survival times were found for patients with both biomarkers highly expressed (Fig. 3C). Thus, for newly diagnosed patients, the combination of high MACC1 and high S100A4 levels improved survival prediction, compared to high MACC1 levels alone.

We then evaluated this combinatorial MACC1 and S100A4 approach with respect to survival for all CRC patients, including the follow-up patients. The cut-off values used were the median of the measured MACC1 levels for all CRC patients (0.518 MACC1 mRNA expression/% calibrator) as well as the median of the measured S100A4 levels for all CRC patients (0.421 S100A4 mRNA expression/% calibrator). In accordance to these cut-off values, all CRC patients were classified as low expressors of both genes, as high expressors of S100A4, as high expressors of MACC1, or as high expressors of both genes. As observed for the newly diagnosed patients, also all CRC patients who showed low circulating levels of MACC1 and of S100A4, had the longest survival times. Expression induction of either MACC1 or S100A4 or of both biomarkers correlated to reduced patients survival (Fig. 3D).

In addition, we also compared the survival of patients with low levels of MACC1 and of S100A4 or with only one marker (either MACC1 or S100A4) increased with the survival of those patients who showed both MACC1 and S100A4 elevated. We found a significantly shorter survival for patients with high plasma levels of both biomarkers. This was observed for the cohort of newly diagnosed patients (*P*<.0001; Fig. S2A) as well as for all CRC patients analyzed (*P* = .001; Fig. S2B).

## Discussion

Here we report the development of the first blood-based assay for the metastasis-inducing gene MACC1, which is a prognostic biomarker for colon cancer metastasis as well as for tumor progression and survival in a variety of solid cancers. This assay for circulating MACC1 transcripts, which can be quantitated on a routine basis, is clinically applicable for diagnosis, prognosis, and therapeutic monitoring of cancer patients. Levels of circulating MACC1 transcripts are significantly higher in cancer patients compared to tumor-free volunteers. Remarkably, highest levels of circulating MACC1 transcripts were determined in individuals with metastases, demonstrating the diagnostic value of circulating MACC1 transcripts in patient plasma with respect to metastasis formation. Most importantly, high MACC1 levels – prospectively determined - correlate with unfavorable survival of the patients underlining the prognostic value of circulating MACC1 transcripts.

Detection of cell-free mRNA in blood as “liquid biopsies” allows real-time monitoring of disease progress, prognosis, and therapeutic response [19]. Numerous studies describe transcript detection of circulating cell-free RNA in plasma and serum of patients suffering from a variety of solid cancers, including thyroid, lung, gastric, breast and uterine cervical cancer, and its use for prognosis of the disease course [20]–[29]. Stability of circulating cell-free RNA is ensured by exosomes (microparticles, microvesicles, multivesicles) protecting it from degradation [30], [31]. Particularly for circulating plasma RNA from colon cancer patients it is reported that this cell-free RNA is confined in mRNA-enriched vesicle-like structures [32]. Several circulating transcripts have already been reported to be useful for diagnosis and prognosis for colorectal cancer; e.g. hTERT [33], β-catenin [34], thymidylate synthase [35], and LISCH7 [36]. Particularly CEA [37], [38], also in combination with markers such as CK19, CK20, hTERT, TIMP-1, miR141 [39]–[41], is recognized as potential aid in early detection of CRC, as prognostic marker for recurrence, and also as predictive biomarker with respect to therapy response. Testing the combination of both, CEA and MACC1, might outperform these features.

Our new findings of circulating MACC1 transcripts in plasma of colorectal cancer patients for diagnosis and prediction of survival are in line with those that we reported previously based on the quantification of mRNA expression in colorectal cancer tissues with respect to disease prognosis [1]. Subsequently, several groups validated the link of high MACC1 expression in colorectal cancer tissue to advanced disease (e.g. [5], [7], [8]). Thus, the employment of the biomarker MACC1 for patient stratification might be extended from tissues to this blood-based assay allowing repeated measurements of circulating MACC1 transcript levels.

The combination of the two metastasis-inducing genes, MACC1 - a key regulator of the HGF/Met signaling pathway [1], with S100A4– a transcriptional target gene of the Wnt/β-catenin signaling pathway [16], improves survival prediction for newly diagnosed colorectal cancer patients. S100A4 has been demonstrated to act as a metastasis-inducing gene [17], [42], [43]. Furthermore, S100A4 has been shown to be a prognostic biomarker when determined in colorectal cancer tissues by qRT-PCR and immunohistochemistry [16], [44]–[48]. We have recently demonstrated the diagnostic and prognostic value of circulating S100A4 transcripts in colorectal cancer and in gastric cancer patients [18]. Thus, combining biomarkers addressing different, but most relevant signaling pathways in colorectal cancer progression enhances the power of patient survival prediction.

The metastasis-inducing MACC1 is a master regulator of the HGF/Met signaling pathway transcriptionally controlling the gene for the receptor tyrosine kinase Met [1], [6], [49], [50], which already was evaluated as therapeutic target in clinical trials [50]. Therefore, MACC1 represents a most promising target for anti-metastatic therapies. In conclusion, this clinically applicable blood-based assay for circulating MACC1 transcripts in cancer patient plasma allows the prognosis of metastasis and survival, but may also prove to be an ideal read-out for monitoring therapeutic response of future interventions targeting MACC1-induced metastasis in cancer patients.

## Supporting Information

Figure S1 **Survival of CRC patients based on circulating S100A4 transcript levels.** Kaplan-Meier analysis for newly diagnosed (A) and all (B) CRC patients, based on S100A4. Patients with high circulating S100A4 transcript levels demonstrated shorter survival (*P* = .112 and *P* = .082, respectively).
(TIF)

Figure S2 **Survival of CRC patients based on circulating transcript levels of a combination of MACC1 and S100A4.** Kaplan-Meier analysis for newly diagnosed (A) and all (B) CRC patients. A. Newly diagnosed CRC patients with low levels of MACC1 and S100A4, or with only one marker (either MACC1 or S100A4) increased (n = 35), had a significantly better survival than patients with both markers elevated (n = 14, *P*<.0001). B. All CRC patients with low levels of MACC1 and S100A4, or with only one marker (either MACC1 or S100A4) increased (n = 209), also demonstrated significantly better survival, when compared to patients with both markers elevated (n = 85, *P* = .001).
(TIF)
