# Supplementary figures and images for: Circulating MACC1 Transcripts in Colorectal Cancer Patient Plasma Predict Metastasis and Prognosis
Source: PLoS One. 2012 Nov 14;7(11):e49249. doi: 10.1371/journal.pone.0049249 (PMC3498161; doi:10.1371/journal.pone.0049249)

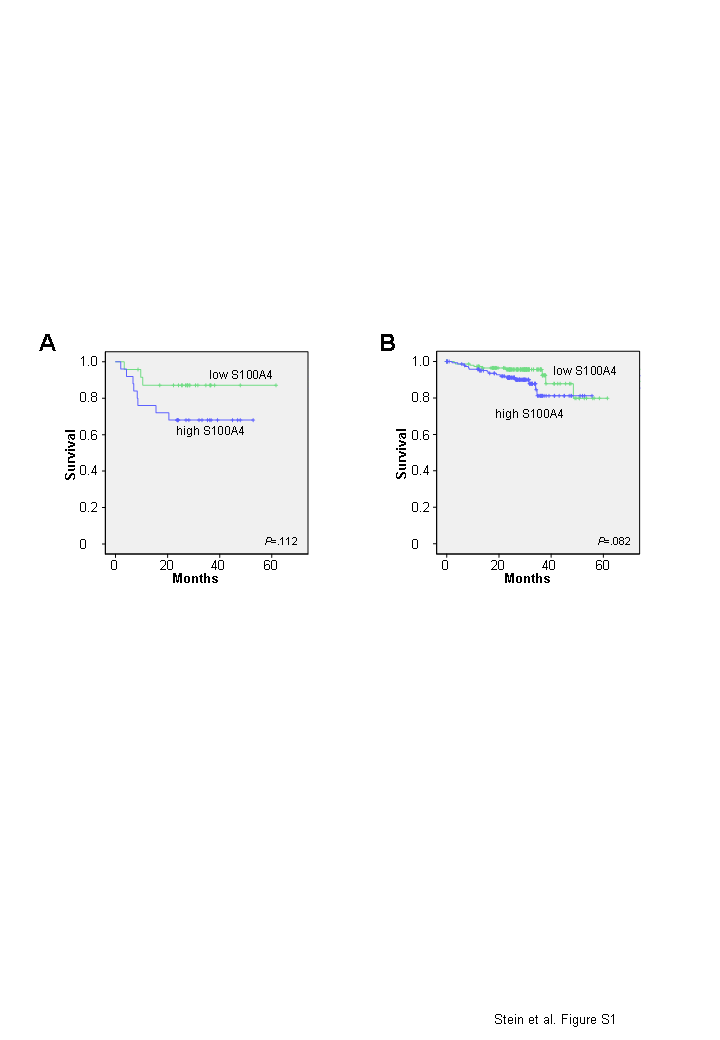

Supplement: Figure S1 — Survival of CRC patients based on circulating S100A4 transcript levels. Kaplan-Meier analysis for newly diagnosed (A) and all (B) CRC patients, based on S100A4. Patients with high circulating S100A4 transcript levels demonstrated shorter survival (P = .112 and P = .082, respectively). (TIF) [file pone.0049249.s001.tif]

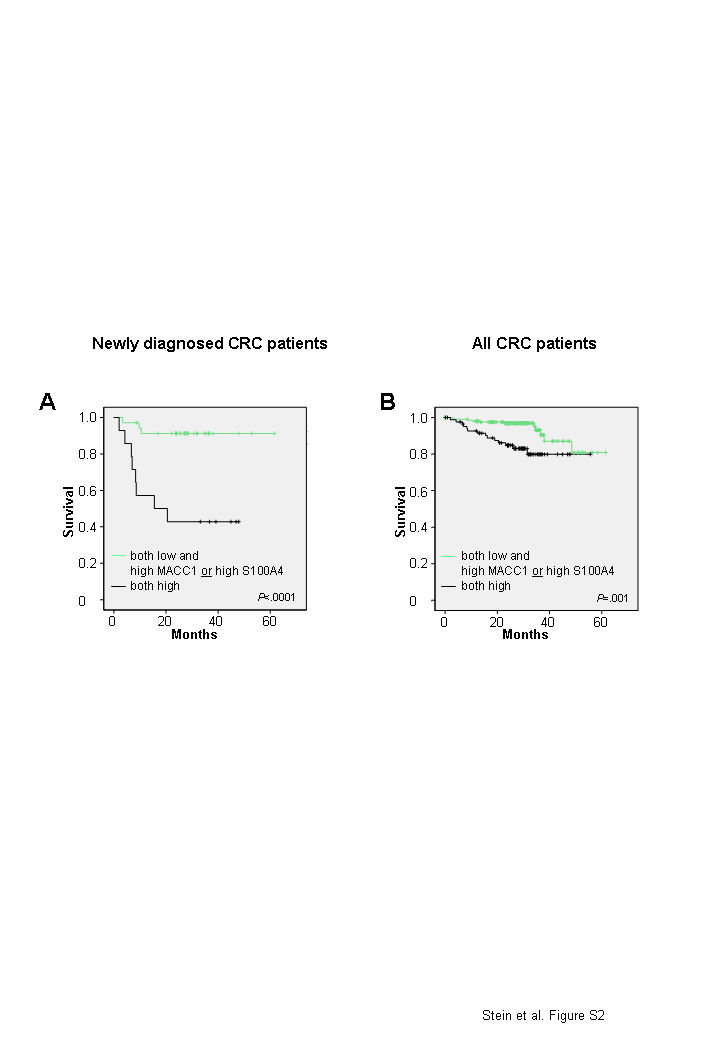

Supplement: Figure S2 — Survival of CRC patients based on circulating transcript levels of a combination of MACC1 and S100A4. Kaplan-Meier analysis for newly diagnosed (A) and all (B) CRC patients. A. Newly diagnosed CRC patients with low levels of MACC1 and S100A4, or with only one marker (either MACC1 or S100A4) increased (n = 35), had a significantly better survival than patients with both markers elevated (n = 14, P<.0001). B. All CRC patients with low levels of MACC1 and S100A4, or with only one marker (either MACC1 or S100A4) increased (n = 209), also demonstrated significantly better survival, when compared to patients with both markers elevated (n = 85, P = .001). (TIF) [file pone.0049249.s002.tif]
